# Fatty acid-binding protein-4 as a biomarker predicting acromegaly-associated diabetes mellitus

**DOI:** 10.3906/sag-2011-317

**Published:** 2021-10-21

**Authors:** Sema HEPŞEN, Pınar AKHANLI, Hakan DÜĞER, Murat ÇALAPKULU, Bekir UÇAN, Muhammed Erkam SENCAR, Davut SAKIZ, İlknur ÖZTÜRK ÜNSAL, Seyit Murat BAYRAM, Mustafa ÖZBEK, Erman ÇAKAL

**Affiliations:** 1 Department of Endocrinology and Metabolism, University of Health Sciences,Dışkapı Yıldırım Beyazıt Training and Research Hospital, Ankara Turkey

**Keywords:** Acromegaly, adipokin, FABP-4, diabetes mellitus

## Abstract

**Background/aim:**

The known pathogenesis of diabetes mellitus (DM) in acromegaly is mainly based on growth hormone (GH) and insulin-like growth factor-1 (IGF-1) excess. Fatty acid-binding protein 4 (FABP-4), a novel adipokine, is found to induce insulin resistance and type 2 DM. We aimed to investigate the possible effect of FABP-4 on glucose metabolism in patients with acromegaly.

**Materials and methods:**

This case-control study included 28 patients newly diagnosed with acromegaly and 57 healthy volunteers. The patients with acromegaly were classified according to their glycemic status as with DM, prediabetes, and normal glucose tolerance. Anthropometric measurements, laboratory test results, and FABP-4 levels of the subjects were evaluated.

**Results:**

Although no difference was observed in FABP-4 levels between acromegaly and control groups, the FABP-4 level was higher in the patients with acromegaly having DM compared to the patients with acromegaly having prediabetes and NGT, and the control group (*p *= 0.004, *p *= 0.001, *p *= 0.004, respectively). Logistic regression analysis suggested that the FABP-4 is an independent predictor of DM in acromegaly (*β *= 7.382, OR = 38.96, 95% CI: 1.52-5.76, *p *= 0.018).

**Conclusion:**

The FABP-4 may be a helpful predictor of acromegaly-associated DM.

## 1. Introduction

Acromegaly develops as a result of excessive growth hormone (GH) and insulin-like growth factor-1 (IGF-1) release and is caused mostly by a pituitary adenoma. The estimated incidence of this rare condition was reported as 0.2–1.1 cases/100000 people/year [1]. Diabetes mellitus (DM), impaired glucose metabolism, insulin resistance, hypertension, and cardiovascular diseases are common in acromegaly and contribute to increased risk of mortality [2,3]. Impaired glucose metabolism is one of the most common metabolic morbidities in acromegaly, and the reported prevalence ranges from 15% to 50% depending on the genetic and ethnic factors of the patients [2]. DM frequency in acromegaly has been indicated in a wide range of 16% to 56% in various studies from different populations [4–6]. 

Despite the impacts of genetic, familial, and environmental risk factors on type 2 DM occurrence, it is known that DM in acromegaly develops as a result of the effects of GH and IGF-1 excess on the pancreatic β-cell function, insulin sensitivity, and gluconeogenesis [7]. Recent studies have revealed that altered adipokine variety and quantity in adipose tissue might contribute to the pathogenesis of insulin resistance and DM in patients with acromegaly [8]. 

Fatty acid-binding protein-4 (FABP-4) is a novel adipokine, which is expressed in adipocytes and plays a part in the initiative mechanism of insulin resistance and type 2 DM via suppressing PPAR gamma [9]. FABP-4 induced PPAR gamma inhibition particularly acts as a role in lipid-mediated inflammation and oxidation processes [9]. With the initiation of this inflammatory process, glycolysis and glucose oxidation begin to be inhibited and the glucose uptake of peripheral tissues, mainly the liver and muscles, reduces [9]. Thus, increased FABP-4 leads to the development of insulin resistance as a consequence of defective insulin secretion. Previous studies have established that FABP-4 was linked to type 2 DM, gestational DM, and metabolic syndrome [10–12]. Although adequate data in the linkage between FABP-4 level and type 2 DM have been presented in the literature, as far as we know, the association between FABP-4 and DM in acromegaly was not investigated before.

 Depending on the hypothesis that FABP-4 was closely relevant to glucose metabolism impairment, we aimed to evaluate FABP-4 levels in patients with acromegaly, especially patients with acromegaly-associated DM.

## 2. Material and methods

### 2.1. Study design 

This study was designed as a case-control study. The Ethics Committee of our institute approved this study regarding the principles of the Helsinki Declaration (Clinical Research Ethics Committee University of Health Sciences, Diskapi Yildirim Beyazit Training and Research Hospital, Approval number: 71/08). Written informed consent was taken before being participated in the study. 

### 2.2. Patients and laboratory tests

Between December 2017 and December 2019, a total of 28 patients were diagnosed with acromegaly in the endocrinology and metabolism department. Acromegaly diagnosis was based on the Endocrine Society Clinical Practice Guideline [13]. Serum IGF-1 level was measured in patients who had typical acromegaly physical features or had acromegaly associated conditions such as type 2 DM, hypertension, and sleep apnea syndrome in the absence of typical acral and facial stigmata. Anthropometric and laboratory data of the patients were examined at the time of diagnosis, before being undergone a pituitary surgery or being started medical treatment with somatostatin analogs. The control group was composed of 57 randomly selected healthy subjects who were admitted to our hospital for routine controls and agreed to participate in our study. 

Foreknown comorbidities of the patients such as hypertension, DM, and atherosclerotic cardiovascular diseases were questioned. DM of the patients with acromegaly was categorized as newly diagnosed or previously known. Diabetic patients were also questioned for their family history of DM. DM and prediabetes diagnosis criteria were based on the American Diabetes Association (ADA) guidelines. DM criteria were based upon either fasting plasma glucose (FPG) ≥ 126 mg/dL or 2-h post-prandial glucose (PG) ≥ 200 mg/dL during oral glucose tolerance test (OGTT) or glycated hemoglobin (HbA1c) ≥ 6.5% (48 mmol/mol) [14]. Prediabetes is defined as the presence of either FPG 100–125 mg/dL or 2-h PG during 75 g OGTT 140–199 mg/dL or HbA1c 5.7%–6.4% (39–46 mmol/mol) [14]. The homeostasis model assessment formula for insulin resistance (HOMA-IR) was evaluated in patients with normal glucose tolerance (NGT) and prediabetes [15]. The patients with acromegaly were divided into subgroups according to their glucose metabolism status in terms of being with DM, prediabetes, and normal glucose tolerance (NGT).

Anthropometric measurements of the participants such as weight, height, waist, and hip circumference were examined. The body mass index (BMI) was calculated by dividing the weight by the square of the height (kg/m^2^). WHO classification was used to determine obesity [16]. Blood pressure was measured from the right arm using a mercury sphygmomanometer with the subject seated after a 10- min rest.

Peripheral blood samples were collected between 8:00 and 10:00 am after at least 8 h of overnight fasting. Serum samples for the FABP-4 analysis were reserved for 2 h at room temperature and then centrifuged for 20 min at approximately 1000×g. All serum samples were stored at −80 °C until the day of analysis. A sandwich enzyme immunoassay kit was used to determine in vitro quantitative measurement of FABP-4 (Cloud-Clone Corp, Houston, TX, USA). FPG was determined through the enzymatic UV test (Beckman Coulter AU, USA). HbA1c level was determined by the ion-exchange high-performance liquid chromatography (HPLC) method (Tosoh Bioscience, G8, USA). Serum lipid parameters were examined using the enzymatic colorimetric method (Beckman Coulter AU, USA). Thyroid-stimulating hormone (TSH), insulin, GH, and IGF-1 levels were analyzed via the chemiluminescence immunoassay method (Beckman Coulter, Access 2, USA). HOMA-IR was calculated according to the following formula: FPG (mg/dL) x fasting insulin (mU/ml)/405. Normal ranges of GH and IGF-1 were as follows: 0.05–8 ng/mL, 42–214 ng/mL, respectively.

Echocardiographic examination was performed using a standard ultrasound system (Philips EPİQ 5, Philips Healthcare, USA). Left ventricle ejection fraction (EF) was evaluated based on the modified biplane Simpson’s method. Left ventricular hypertrophy (LVH) was defined as an increase in the size of myocardial fibers of the left ventricle [17].

### 2.3. Statistical analysis

Statistical analyses were performed using the SPSS software version 21 (Chicago, IL). The variables were investigated by means of visual (histograms, probability plots) and analytic methods (Kolmogorov–Smirnov/Shapiro–Wilk’s test) to determine whether they are normally distributed or not. While the Student’s t-test was used to compare age, waist circumference, creatinine, total cholesterol, high-density lipoprotein cholesterol (HDL-C), and low-density lipoprotein cholesterol (LDL-C) levels, the Mann–Whitney U test was performed to compare sex, BMI, hip circumference, systolic and diastolic blood pressure, HOMA-IR, FPG, HbA1c, GH, IGF-1, TSH, ALT, and triglyceride levels. Descriptive analyses were presented using means and standard deviations for normally distributed variables, whereas medians and interquartile ranges (IQR) between 25 and 75 percentiles for nonnormally distributed variables. A *p*-value less than 0.05 was considered to show a statistically significant difference. The Kruskal-Wallis tests were conducted to compare FABP-4 levels and other laboratory parameters among the groups, which were composed according to their glucose metabolism and insulin resistance status. The Mann–Whitney U test was performed to examine the significance of pairwise differences using Bonferroni correction to adjust for multiple comparisons. Overall, a 5% type-1 error level was used to infer statistical significance. While investigating the associations between FABP-4 level and other variables, correlation coefficients and their significance were calculated using the Spearman test. A logistic regression analysis was used to identify the independent predictors of DM in acromegaly. Hosmer–Lemeshow goodness of fit statistics was used to assess model fit. A 5% type-I error level was used to infer statistical significance. 

## 3. Results

Twenty-eight patients newly diagnosed with acromegaly and 57 controls were included in the study. The age and sex distribution of the controls were similar to the patients with acromegaly (*p *> 0.05). The patients with acromegaly had higher BMI and waist circumference than controls (*p *= 0.014, *p *= 0.02 respectively). All of the patients with acromegaly had a pituitary adenoma. Five (17.8%) patients had a microadenoma, while 23 (82.2%) had a macroadenoma. 26 (92.8%) patients underwent a transnasal transsphenoidal pituitary surgery, and 2 (7.2%) patients were started on somatostatin analog treatment as the first-line therapy. A total of 46.4% of patients with acromegaly had hypertension. Systolic and diastolic blood pressure values were higher in the patients with acromegaly than in the control group (*p *= 0.001 and *p* < 0.001, respectively). Nine of 28 patients with acromegaly (32.1%) had DM and 9 of 28 (32.1%) had prediabetes. Only one patient with acromegaly had a family history of DM. While 5 of 9 diabetic patients were diagnosed with DM and acromegaly concurrently, 4 patients had a previously diagnosed DM. The initial treatment was a pituitary surgery for all patients with acromegaly with DM and prediabetes, and glucose metabolism was improved in all of these patients after the surgery. Five of 28 patients with acromegaly (17.8%) had insulin resistance. FPG, HbA1c levels, HOMA-IR, GH, and IGF-1 were higher in the patients with acromegaly than that of controls (*p* < 0.0001 for each). Baseline characteristics and laboratory test results belonging to the groups are presented in Table 1. 

**Table 1:** The comparisons of demographic data, anthropometric measurements, and laboratory test results of the patients with acromegaly and controls.

	Patients with acromegaly	Controls	p value
Demographic and anthropometric data			
Number, n	28	57	
Age, years	48.2 ± 8.8	50.3 ± 10.3	.319
Female, n (%)	18 (64.3)	40 (70.2)	.586
BMI, kg/m2	30.4 (27.6–35.6)	28.4 (25–31.8)	.014
Waist circumference, cm	98.8 ± 15.3	89.3 ± 9.7	.02
Hip circumference, cm	103 (100–120)	105 (100–110)	.446
Systolic BP, mm/Hg	120 (120–130)	120 (110–120)	.001
Diastolic BP, mm/Hg	75 (70–80)	70 (60–70)	< .0001
Hypertension, n (%)	13 (46.4)	0 (0)	-
Diabetes mellitus, n (%)	9 (32.1)	0 (0)	-
Prediabetes, n (%)	9 (32.1)	0 (0)	-
Insulin resistance, n (%)	5 (17.8)	0 (0)	-
Obesity, n (%)	15 (53.6)	21 (36.8)	.145
LVH, n (%)	8 (28.7)	0 (0)	.014
EF, %	65 (60–65)	65 (60–65)	.888
Laboratory test results			
FPG, mg/dL	103 (91–138)	83 (80–90)	< .0001
HbA1c, %	6.1 (5.7–7)	5.6 (5.4–5.7)	< .0001
HOMA–IR	3.7 (1.9–6.4)	1.15 (0.9–1.6)	< .0001
Total cholesterol, mg/dl	187 ± 19	188 ± 42	.889
Triglyceride, mg/dl	128 (109–189)	99 (68–138)	.005
HDL-C, mg/dL	43 ± 8	50 ±10	.002
LDL-C, mg/dL	129 ± 19	132 ± 35	.669
Creatinine, mg/dL	0.79 ± 0.2	0.83 ± 0.8	.076
ALT, mg/dL	15 (14–18)	18 (15–23)	.088
TSH, µIU/mL	1 (0.6–1.8)	1.3 (1–2)	.084
IGF-1, ng/mL	536 (453–629)	169 (133–194)	< .0001
GH, ng/mL	10.2 (3.4–35)	1.9 (1.45–2.3)	< .0001
FABP-4, ng/mL	1.52 (1.42–1.81)	1.61 (1.49–1.87)	.286

Categorical data were demonstrated with numbers and percentages (%). Normally distributed variables were presented as means (standard deviations) and nonnormally distributed variables were presented as medians (interquartile range: 25–75).Abbreviations: BMI: body mass index, BP: blood pressure, LVH: left ventricle hypertrophy, EF: ejection fraction, FPG: fasting plasma glucose, HDL-C: high-density lipoprotein cholesterol, LDL-C: low-density lipoprotein cholesterol, ALT: alanine aminotransferase, TSH: thyroid-stimulating hormone, IGF-1: insulin-like growth factor-1, GH: growth hormone, FABP-4: fatty acid-binding protein 4.

The difference in FABP-4 levels between all patients with acromegaly and the control group was not statistically significant (*p* = 0.286). A statistically significant difference was observed in FABP-4 levels among the groups when compared the patients with acromegaly having DM and the controls, with acromegaly having DM and prediabetes, with acromegaly having DM and NGT, respectively (*p* = 0.014, *p* = 0.004, *p* = 0.001) (Figure). Despite the difference was not statistically significant, the patients with acromegaly having DM had higher levels of GH compared to the patients with acromegaly having prediabetes and NGT (*p* = 0.077, *p* = 133, respectively). Baseline data, laboratory parameters, and FABP-4 levels of the groups are presented in Table 2. 

**Table 2:** The comparisons of baseline characteristics and laboratory parameters among acromegaly groups in terms of their glucose metabolism statuses and controls

	Acromegaly with DM	Acromegaly with prediabetes	Acromegalywith NGT	Controls	p valueDM vs controls	p valueDM vs prediabetes	p valueDM vs NGT
Number, n (%)	9 (32.1)	9 (32.1)	10 (35.8)	57 (100)			
Female/male	5/4	5/4	8/2	40/17	0.385	1.00	0.4
BMI, kg/m2	29.2 (28–31.7)	36.8 (28–42)	30.5 (25.6–33.6)	28.4 (25–31.8)	0.318	0.149	0.963
FABP-4, ng/mL	2 (1.71–2.23)	1.47 (1.42–1.55)	1.47 (1.34–1.58)	1.6 (1.49–1.87)	0.014	0.004	0.001
FPG, mg/dL	175(130–189)	104 (99–106)	90 (82–95)	83 (80–90)	<0.001	<0.001	<0.001
HbA1c, %	9.8 (7.5–12.4)	6.1 (6–6.3)	5.6 (5.6–5.7)	5.6 (5.4–5.7)	<0.001	<0.001	<0.001
IGF-1, ng/mL	507 (421–632)	545 (442–787)	536 (446–602)	169 (133–194)	<.0001	0.077	0.133
GH, ng/mL	21 (9.4–40)	8 (3–18.3)	5.5 (1.7–24.5)	1.9 (1.45–2.3)	<.0001	0.436	0.842

Nonnormally distributed variables were presented as medians (interquartile range: 25–75).Abbreviations: FABP-4: fatty acid-binding protein 4, DM: diabetes mellitus, NGT: normal glucose tolerance, BMI: body mass index, FPG: fasting plasma glucose, IGF-1: insulin-like growth factor-1, GH: growth hormone.

**Figure:**
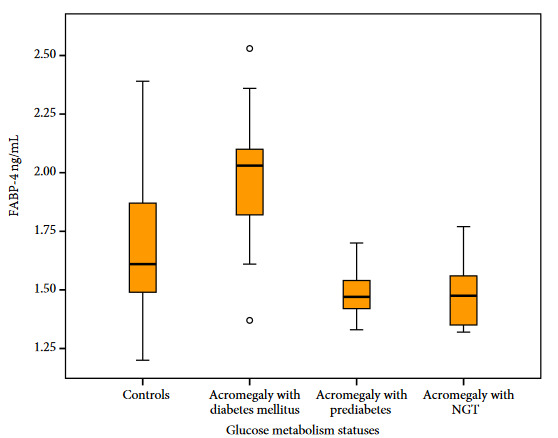
Estimated FABP4 levels according to glucose metabolism statuses. The p values were as follows among groups: p = 0.004 (acromegaly with diabetes vs. acromegaly with prediabetes), p = 0.001 (acromegaly with diabetes vs. acromegaly with normal glucose tolerance), p = 0.014 (acromegaly with diabetes vs. controls).

No difference was observed in FABP-4 levels compared to the patients with and without insulin resistance (*p* = 0.380), obesity (*p* = 0.223), hypertension (*p* = 0.995), and LVH (*p* = 0.553).

We established that the FABP-4 is positively correlated to FPG, HbA1c, and GH levels in the acromegaly group (*r *= 0.548; *p* = 0.003,* r *= 0.478; *p* = 0.012, *r *= 0.418; *p* = 0.027, respectively). However, any association is not determined between FABP4 levels and age, sex, BMI, FPG, HbA1c, GH, and IGF-1 in the whole study group including both patients and controls. Logistic regression analysis was calculated to detect independent predictors of DM in acromegaly based on the variables, including age, sex, FABP-4, IGF-1, and GH levels and was presented in Table 3. Logistic regression analysis suggested that the FABP-4 level is an independent predictor of DM but not the other variables (*β *= 7.382, OR = 38.96, 95% CI: 1.52-5.76, *p* = 0.018).

**Table 3:** Binary logistic regression analysis results showing the predictive values of the parameters for the presence of diabetes mellitus.

	B	SE	OR	95% CI	p value
Age	0.186	0.117	0.111	0.95–1.51	0.953
Sex	0.079	1.33	1.082	0.1–14.7	0.953
GH	0.102	0.058	1.107	0.98–1.24	0.077
IGF-1	0.005	0.004	1.005	0.99–1.01	0.243
FABP-4	7.382	3.11	38.96	1.52–5.76	0.018

Abbreviations: SE: standard error, OR: odds ratio, CI: confidence interval, IGF-1: insulin-like growth factor-1, GH: growth hormone, FABP-4: fatty acid-binding protein 4.

## 4. Discussion

The results of the present study demonstrated that FABP-4 levels were higher in the patients with acromegaly having DM compared to the patients without DM and the controls. Logistic regression analysis also suggested that the FABP-4 level is an independent predictor of DM in acromegaly but not age, sex, IGF-1, and GH.

Patients with acromegaly have a higher risk of glucose metabolism disorders compared to the general population [5]. While DM prevalence in the patients with acromegaly varies in the range of 16% to 56% among different study populations, the prevalence of impaired fasting glucose and glucose tolerance have been reported as 8.9% to 19% and 15% to 31.6%, respectively [5,6,18]. Similar to the literature, the frequencies were found out 32.1% for DM and 32.1% for prediabetes in the present study.

GH physiologically affects glucose metabolism and leads to hepatic gluconeogenesis, lipolysis, and glycogenolysis. Increased glucose and free fatty acid production induce insulin resistance in the liver and peripheral tissues [19]. Besides the direct effects of GH contrary to insulin action, GH conduces to improve insulin activity via stimulating IGF-1 [7]. However, the insulin agonistic effect of IGF-1 is inadequate to overcome the direct impact of GH on glucose metabolism. Impaired glucose metabolism and DM develop as a result of decreased insulin sensitivity and secretion [20]. 

Adipose tissue, one of the targets of GH excess, plays a significant role in glucose metabolism impairment via lipolytic activity [7]. Previous studies have established that GH increases adipose tissue hormone-sensitive lipase activity and leads to FFA release to the peripheral tissues [7]. This was assumed that adipose tissue dysfunction is more important than the amount of the accumulated visceral fat in the pathogenesis of insulin resistance and DM in acromegaly [19]. Furthermore, the adipose tissue dysfunction changes the variety and quantity of adipokines and a change in adipokine distribution causes a more inflamed fat that is suspected in insulin resistance and DM [8]. These novel studies bring to mind that the occurrence of DM in acromegaly may not be solely mediated with GH.

The FABP-4 is a member of the calycin protein superfamily and is expressed in adipocytes and macrophages [9]. FABP-4 expression particularly enhances when the adipocytes were differentiating [9]. FABP-4 acts a crucial role in glucose and lipid homeostasis through mediating inflammation and oxidation. The FABP-4 affects glucose metabolism via negatively regulating PPAR gamma activity, which promotes adipocyte differentiation, proliferation, and insulin sensitivity of adipocytes [21,22]. With the activation of this inflammatory process, the inhibition of glucose oxidation and glycolysis begins, and glucose uptake of peripheral tissues such as the liver and muscles decreases [9]. FABP-4 also contributes to insulin resistance by accumulating short-chain-free fatty acids. Previous studies have established that FABP-4 is associated with type 2 DM, gestational DM, cardiovascular diseases, obesity, and metabolic syndrome [23–25]. A 10-year study including the Chinese population has demonstrated that the FABP-4 level is a predictor of DM development [26]. Another study including the U.S. male participants have determined that the FABP-4 level is associated with the increased risk of DM [12]. In this study, there was no difference in FABP-4 levels among the groups with and without obesity, hypertension, and LVH. According to the results of the present study, the FABP-4 level was found to be higher in patients with acromegaly and DM. Only one patient had a family history of DM in our diabetic acromegaly group. DM was diagnosed concurrently with acromegaly in most of the patients. Glucose metabolism of all patients with DM and prediabetes was improved after the surgery as well. All these findings taken together indicate that the diagnosis of DM of our patients with acromegaly was related to acromegaly rather than concomitant type 2 DM to acromegaly. This is noteworthy to point that the correlation analysis of this study showed a linear association between FABP-4 level and HbA1c in the acromegaly group. The patients with acromegaly having DM have also a higher HbA1c than the other 3 groups. With respect to these results, it can be commentated that a higher FABP-4 level may indicate poorly controlled DM. However, there was no difference between our patients with and without insulin resistance. The small sample size belonging to subgroups may be the explanation for this discrepancy.

This was also shown that GH modulates the expression of gene-related adipocyte differentiation and inhibits the expression of various molecules including adiponectin and PPAR gamma [27]. In the present study, the patients with acromegaly having DM had higher GH levels compared to the patients with acromegaly having prediabetes and NGT, and FABP-4 was found positively correlated to GH. It may be hypothesized that GH may also be mediating FABP-4 expression because they both induce PPAR gamma inhibition. 

Various studies have indicated that higher levels of IGF-1, older age, and family history of DM were related to DM in acromegaly [5,28]. A study evaluating the French acromegaly population has suggested that age, BMI, and hypertension were predictors of DM presence, but not IGF-1 and GH [6]. IGF-1 level at the diagnosis time and the presence of hypertension were found to be associated with DM development in another study including Mexican patients with acromegaly [29]. Our study revealed that the FABP-4 is an independent predictor of DM.

That having a small size of samples in groups is a limitation of our study. However, the number of patients may be acceptable considering the rarity of acromegaly. We also included all patients newly diagnosed with acromegaly in our institute within the defined period to eliminate any bias. A point that needs to be clarified is that the obesity rate of control subjects is close to the rate of the patients with acromegaly. However, controls were selected randomly and we did not determine any relationship between obesity and FABP-4 levels.

In conclusion, the FABP-4 level was determined to be higher in the patients with acromegaly having DM and it may be a helpful predictor of acromegaly-associated DM. 

## Informed consent 

The Ethics Committee of our institute approved this study regarding the principles of the Helsinki Declaration (Clinical Research Ethics Committee University of Health Sciences, Diskapi Yildirim Beyazit Training and Research Hospital, Approval number: 71/08). Written informed consent was taken from all participants before being participated in the study. 

## Availability of data and materials 

The datasets used and/or analyzed during the current study are available from the corresponding author on reasonable request.

## Contribution of authors 

SH, PA, ES, DS, and EC contributed to the study conception and design. SH, PA, IU, DS, ES, BU, SMB, MC, and HD performed material preparation and data collection. SH performed the statistical analyses. SH wrote the first draft of the manuscript. EC and MO supervised the study. All authors commented on the manuscript. All authors read and approved the final manuscript.
